# Supplementary material for: Exploring the Link Between Physical Activity, Sports Participation, and Loneliness in Adolescents Before and Into the COVID-19 Pandemic: The HUNT Study, Norway
Source: Int J Environ Res Public Health. 2024 Oct 25;21(11):1417. doi: 10.3390/ijerph21111417 (PMC11594226; doi:10.3390/ijerph21111417)
Supplement: Supplementary file 1 [file ijerph-21-01417-s001.zip › ijerph-3179560-supplementary.pdf]

Supplement S1:

Table S1. Odds ratios (OR) with 95% confidence intervals (CIs) for the probability of loneliness through four years from YH1 to YH2 (Cohort 1, n=2066) measured by stable loneliness, increasing or decreasing loneliness compared to not being lonely predicted by physical activity and sport participation stratified on gender.

	Outcome								
	Model 1			Model 2			Model 3		
	Lonely OR (95% CI)	Decreasing loneliness OR (95% CI)	Increasing loneliness OR (95% CI)	Lonely OR 95% CI	Decreasing loneliness OR 95% CI	Increasing loneliness OR 95% CI	Lonely OR 95% CI	Decreasing loneliness OR 95% CI	Increasing loneliness OR 95% CI
Girls									
Physical activity									
Active (≥150min/wk)	1.0	1.0	1.0	1.0	1.0	1.0	1.0	1.0	1.0
Inactive (<150min/wk)	1.57** (1.11-2.21)	1.30 (0.89-1.90)	1.56** (1.12-2.17)	1.45* (1.02-2.08)	1.29 (0.87-1.90)	1.45* (1.03-2.03)	1.45 (0.98-2.12	1.20 (0.80-1.81)	1.45* (1.03-2.05)
Boys									
Physical activity									
Active (≥150min/wk)	1.0	1.0	1.0	1.0	1.0	1.0	1.0	1.0	1.0
Inactive (<150min/wk)	1.55 (0.59-2.51)	1.39 (0.89-2.18)	1.87** (1.25-2.78)	1.34 (0.81-2.22)	1.38 (0.87-2.19)	1.70** (1.12-2.58)	1.16 (0.66-2.01)	1.34 (0.84-2.16)	1.78** (1.17-2.71)
Girls									
Sport participation									
No	1.0	1.0	1.0	1.0	1.0	1.0	1.0	1.0	1.0
Yes	.58*** (0.42-0.81)	.57*** (0.40-0.82)	.79 (0.57-1.09)	.61** (0.43-0.85)	.59* (0.41-0.85)	.82 (0.59-1.13)	.68* (0.47-0.98)	.65* (0.44-0.95)	.84 (0.60-1.18)
Boys									
Sport participation									
No	1.0	1.0	1.0	1.0	1.0	1.0	1.0	1.0	1.0
Yes	.52** (0.34-0.80)	.81 (0.53-1.22)	.68* (0.46-0.99)	.54* (0.34-0.85)	.82 (0.54-1.26)	.73 (0.49-1.08)	.65 (0.40-1.08)	.91 (0.59-1.43)	.70 (0.47-1.05)

Model 1 - Associations adjusted for age and stratified by sex in YH1.
Model 2 - Associations adjusted for age, educational aspirations, visiting friends occurrence and stratified by sex in YH1.
Model 3 - Fully adjusted model. All risk factors and covariates (baseline (YH1) values for: educational aspirations, visiting friends occurrence, self-rated health, and mental distress (HSCL-5)) are included in the model in addition to age and sex.
ORs Odds Ratios, CI Confidence Interval, * $p < 0.05$, ** $p < 0.01$, *** $p < 0.001$
Not lonely = not lonely in YH1 and YH2, Lonely = lonely in YH1 and YH2, Decreasing loneliness = changing loneliness category from lonely in YH1 to not lonely in YH2, Increasing loneliness = not lonely in YH1 and lonely in YH2

Table S2. Odds ratios (OR) with 95% confidence intervals (CIs) for the probability of loneliness through four years from YH4 to YHC (Cohort 1, n=1565) measured by stable loneliness, increasing or decreasing loneliness compared to not being lonely predicted by physical activity and sport participation stratified on gender.

	Outcome								
	Model 1			Model 2			Model 3		
	Lonely OR (95% CI)	Decreasing loneliness OR (95% CI)	Increasing loneliness OR (95% CI)	Lonely OR 95% CI	Decreasing loneliness OR 95% CI	Increasing loneliness OR 95% CI	Lonely OR 95% CI	Decreasing loneliness OR 95% CI	Increasing loneliness OR 95% CI
Girls									
Physical activity									
Active (≥150min/wk)	1.0	1.0	1.0	1.0	1.0	1.0	1.0	1.0	1.0
Inactive (<150min/wk)	2.05***	0.92	1.32	2.26*	1.09	1.50	1.22	0.71	1.42
	(1.35-3.08)	(0.52-1.63)	(0.91-1.94)	(1.37-3.72)	(0.55-2.14)	(0.95-2.38)	(0.67-2.23)	(0.33-1.5)	(0.86-2.32)
Boys									
Physical activity									
Active (≥150min/wk)	1.0	1.0	1.0	1.0	1.0	1.0	1.0	1.0	1.0
Inactive (<150min/wk)	4.06***	2.39*	2.21**	4.74***	2.11	2.91***	3.13*	2.02	2.55*
	(2.07-7.97)	(1.08-4.20)	(1.32-3.68)	(2.19-10.22)	(0.96-4.62)	(1.61-5.26)	(1.29-7.61)	(0.86-4.72)	(1.36-4.78)
Girls									
Sport participation									
No	1.0	1.0	1.0	1.0	1.0	1.0	1.0	1.0	1.0
Yes	.48***	.99	.68	0.85	1.33	.70	1.09	1.85*	.79
	(0.32-0.72)	(0.58-1.71)	(0.47-0.98)	(0.48-1.49)	(0.66-2.66)	(0.44-1.12)	(0.65-1.82)	(1.00-3.41)	(0.53-1.17)
Boys									
Sport participation									
No	1.0	1.0	1.0	1.0	1.0	1.0	1.0	1.0	1.0
Yes	.28***	.80	.60*	.40*	1.06	.60	.44*	0.99	.75
	(0.14-0.54)	(0.40-1.60)	(0.37-0.98)	(0.16-0.95)	(0.44-2.53)	(0.32-1.10)	(0.20-0.97)	(0.43-2.25)	(0.44-1.29)

Model 1 - Associations adjusted for age in YH1 and sex.
Model 2 - Associations adjusted for age, educational aspirations, visiting friends occurrence and stratified by sex in YH1.
Model 3 - Fully adjusted model. All risk factors and covariates (baseline (YH1) values for: educational aspirations, visiting friends occurrence, self-rated health, and mental distress (HSCL-5)) are included in the model in addition to age and sex.
ORs Odds Ratios, CI Confidence Interval, * $p < 0.05$, ** $p < 0.01$, *** $p < 0.001$
Not lonely/Lonely = self-reported lonely in YH4 and YHC, Decreasing loneliness = lonely YH4 and not lonely YHC, Increasing loneliness = not lonely YH4 and lonely YHC.
